# Electrocardiographic Parameters Associated with Adverse Outcomes in Children with Cardiomyopathies

**DOI:** 10.3390/jcm11236930

**Published:** 2022-11-24

**Authors:** Katarzyna Luczak-Wozniak, Klaudia Obsznajczyk, Cezary Niszczota, Bożena Werner

**Affiliations:** 1 Department of Pediatric Cardiology and General Pediatrics, Doctoral School, Medical University of Warsaw, 02-091 Warsaw, Poland; 2 Department of Pediatric Cardiology and General Pediatrics, Jozef Polikarp Brudzinski Public Pediatric Hospital, 02-091 Warsaw, Poland; 3 Department of Pediatric Cardiology and General Pediatrics, Medical University of Warsaw, 02-091 Warsaw, Poland

**Keywords:** hypertrophic cardiomyopathy, dilated cardiomyopathy, left ventricular non-compaction, QRS-T angle, electrocardiography, pediatric, children

## Abstract

Cardiomyopathies have a low prevalence in children and thus may lead to malignant ventricular arrhythmias or the progression of heart failure, resulting in death. In adults, the QRS-T angle derived from ECG has been associated with adverse outcomes in patients with hypertrophic and dilated cardiomyopathies. We aimed to assess the electrocardiographic parameters, including QRS-T angle, associated with adverse cardiac events in children with cardiomyopathies. Forty-two children with cardiomyopathies were included in this study: 19 with dilated cardiomyopathy, 17 with hypertrophic cardiomyopathy, and 6 with left ventricular non-compaction. Additionally, 19 control subjects were recruited. In terms of ECG parameters, the QRS-T angle was significantly greater among patients with adverse outcomes compared to patients without the end points of the study (133° vs. 65°, *p* < 0.001). On Kaplan–Meier survival curves, QRS-T angle > 120°, increased serum concentrations of NT-proBNP and troponin I levels as well as greater NYHA or Ross scale were associated with the greatest risk of unfavorable outcome. The QRS-T angle appears to be a valuable component of 12-lead ECG interpretation, and might be helpful in outlining patients with the greatest cardiovascular risk. Additionally, serum biomarkers such as NT-proBNP (*p* = 0.003) and troponin (*p* < 0.001) are useful in outlining patients with the worst survival.

## 1. Introduction

Vectorcardiography (VCG) is a field of electrocardiography that describes the direction and magnitude of the electrical forces conducted between cardiomyocytes [1]. The QRS-T angle can be calculated between the vector of depolarization (QRS-complex) and repolarization (T wave) providing information about the myocytes’ electrical conductivity and action potential’s heterogeneity [1]. Abnormal conduction will result in an increased QRS-T angle, even though standard 12-lead ECG (electrocardiogram) may not show any specific abnormalities.

In adults with non-ischemic dilated cardiomyopathies, a greater QRS-T angle is a risk factor for ventricular arrhythmias, re-hospitalizations due to heart failure, and increased mortality [2,3,4]. A similar observation was made in patients with hypertrophic cardiomyopathy (HCM) in whom a wider QRS-T angle was associated with greater risk for ventricular arrhythmia [5,6]. Furthermore, an association between some genetic mutations (LZTR1, LGMD, SCN5a) and ion channel abnormalities of the myocytes was shown for cells with dilated and hypertrophic cardiomyopathies [7,8,9]. Electrical instability of the myocardium, possibly due to genetic predisposition leading to ion channel instability, fibrosis, or abnormal stretching of the myocardial cells, can be assessed with a greater QRS-T angle. 

It is of note that ventricular tachycardia in children may also be caused by channelopathies. Models helpful in understanding the pathophysiology and treatment of SQTS are being developed [10]. Furthermore, cell studies shine new light on optimizing pharmacotherapy in SQTS and Brugada Syndrome [11,12]. Considering the growing understating of pathophysiology and treatment of channelopathies, they should be excluded in all children with ventricular tachycardia, especially, that overlapping of cardiomyopathy and channelopathy genes has been reported [13]. In all hypertrophic, dilated and left ventricular non-compaction cardiomyopathies, ventricular arrhythmia may occur and heart failure may progress, resulting in death. The literature on the usefulness of the QRS-T angle in children with hypertrophic and non-compaction cardiomyopathies is limited [14,15]. To our knowledge, the QRS-T angle in children with idiopathic dilated cardiomyopathy (DCM) has not been assessed previously.

In our study, we focused on hypertrophic, dilated and left ventricular non-compaction cardiomyopathies because they are the most common among children [16]. We hypothesized that repolarization and depolarization abnormalities assessed by QRS-T angle may help to predict not only arrhythmia but also abnormal ventricular function, heart transplant qualification, or death. We aimed to assess electrocardiographic factors associated with unfavorable outcomes of pediatric dilated, hypertrophic, and left ventricular non-compaction cardiomyopathies.

## 2. Materials and Methods

In this single-center prospective study, we included 42 children with cardiomyopathies: 19 with idiopathic dilated cardiomyopathy (DCM), 17 with hypertrophic cardiomyopathy (HCM) and 6 with left ventricular non-compaction (LVNC). Additionally, 19 healthy, gender- and age-matched subjects were recruited. 

The diagnosis of cardiomyopathy was stated on the basis of echocardiography; in borderline cases cardiac magnetic resonance imaging (CMR) was used for verification. Dilated cardiomyopathy was defined as enlargement of the left ventricle with a *z*-score > 2 combined with reduced ejection fraction (<55%). Secondary causes such as: aortic stenosis, coarctation of the aorta, or anomalous coronary arteries were excluded in all patients [16]. Hypertrophic cardiomyopathy was diagnosed according to the ESC 2014 criteria and defined as a wall thickness *z*-score > 2 [17]. Left ventricular non-compaction cardiomyopathy was identified using criteria from Jenni et al., i.e., non-compaction to compaction ratio > 2 in systole [18].

The control group consisted of healthy children with normal echocardiographic examinations. The exclusion criteria of participating in the study were co-existing congenital heart defects, syndromic cardiomyopathies, co-existing chronic diseases, dysmorphic features, and lack of consent to participate in the study.

According to the study protocol, all patients had a 12-lead electrocardiogram (ECG), 24 h ECG Holter monitoring, and echocardiography performed at the beginning of the study. Additionally, serum concentrations of cardiac biomarkers (highly sensitive troponin I and N-terminal prohormone of brain natriuretic peptide (NT-proBNP)) were measured in children with cardiomyopathies. Symptoms of heart failure were assessed using the NYHA scale or Ross scale in younger children [19]. Data concerning the previous history of ventricular tachycardia were also collected. An unfavorable outcome of the disease was defined as a major adverse cardiac event (MACE) and included the following: cardiac death, qualification for heart transplantation, left-ventricular assist device (LVAD) implantation, or the occurrence of ventricular tachycardia. Furthermore, the end points of the study were divided into heart failure end points (death due to progression of heart failure, qualification for heart transplantation) and arhythmic end points (ventricular tachycardia, sudden cardiac death, qualification for an ICD (implantable cardioverter defibrillator) implantation).

The study was approved by the University Bioethical Committee. It was registered at ClinicalTrial.gov (NCT04316923). All parents or guardians, as well as patients aged 16 years or older, signed a written informed consent form prior to participating in the study.

### 2.1. Electrocardiography (ECG)

Twelve-channel electrocardiograms were performed using 50 mm/s speed and 10 mm/mV amplitude for limb and precordial leads. The electrocardiograms were analyzed by 2 independent observers, and discrepancies were resolved by a third reviewer. In each electrocardiogram heart rhythm, heart rate, heart axis, atrial and ventricular hypertrophy, duration of: PQ, QRS, QT and QTc intervals, Q wave abnormalities, ST changes, T wave abnormalities, and spatial QRS-T angle were assessed. QTc was calculated using Bazzett’s formula.

The spatial QRS-T angle was assessed according to the previously described Kors’ quasi-orthogonal transform method using visual estimation of the limb and precordial leads [20,21]. Patients with bundle branch blocks were excluded from the QRS-T analysis [21].

### 2.2. Echocardiography

Echocardiography was performed using a Philips EPIQ ultrasound system 9.0.1 (Koninklijke Philips N.V., Amsterdam, The Netherlands) with X5-1, S5-2, and S8-3 transducers. The left ventricular ejection fraction (LVEF) was assessed using Simpson’s method. The left ventricular internal diastolic diameter (LVIDd) and left ventricular posterior wall thickness in diastole (LVPWd) were evaluated using M-mode; *z*-scores were used to adjust for differences in body weight and height [22].

### 2.3. Statistical Analysis

Continuous variables with normal distribution are presented as mean and standard deviation (SD); when skewed distribution is present the median and inter quartile range (IQR) are given. Categorical variables are presented as frequencies and percentages. The statistical analysis was performed using R version 4.1.2 (1 November 2021), GNU General Public License. Continuous variables were compared using T-test, Mann–Whitney U test, F test, and Kruskal–Wallis test, depending on the number and distribution of the compared variables. Categorical variables were compared using the Chi-Square test.

Correlations were assessed using Pearson’s or Spearman’s coefficient, depending on the distribution of variables. A random forest model was used to outline the most useful parameters in predicting adverse outcomes in all cardiomyopathy patients as well as in the HCM and DCM groups. Due to the limited number of patients included in the study Kaplan–Meier survival analysis was calculated including all cardiomyopathy patients. The factors associated with unfavorable outcomes could not be calculated separately for the LVNC group due to a limited number of patients.

Lin’s correlation coefficient was used to compare the accuracy of the ECG measurements between 2 observers. A *p*-value < 0.05 was considered statistically significant.

## 3. Results

Forty-two children with cardiomyopathies (19 with DCM, 17 with HCM, 6 with LVNC) and 19 healthy control subjects were included in the study. The median age among children with cardiomyopathies was 10 years (IQR 3–15 years), there were 23 females, and the mean time of observation was 13 months. There were no significant differences in terms of gender, age, and BSA between the control subjects and the HCM, DCM and LVNC patients. The groups’ characteristics are presented in Table 1.

Baseline 12-lead ECGs were not significantly different among DCM, HCM, and LVNC patients in terms of heart rate, PQ, QRS, and QTc intervals as well as QRS-T angle (Table S1 Supplementary Materials). None of the patients presented with short QTc interval. Based on genetic testing and clinical investigation no one fulfilled criteria for LQTS. 

When compared to the control group, DCM patients did not differ in terms of heart rate, PQ, QRS, and QTc interval; however, they presented with a wider QRS-T angle (75° vs. 41° *p* = 0.007). In the HCM group heart rate, PQ and QRS intervals were not significantly different from the control group. Thus, a greater mean QTc interval (420 vs. 380 ms *p* = 0.002), QRS-T angle (100° vs. 41° *p* < 0.001), and two or more negative T waves (*p* = 0.005) were observed more frequently in HCM patients than in the control group. The LVNC group did not differ from the control group in terms of heart rate, PQ, and QRS duration. Thus, a greater QTc interval (420 vs. 380 ms *p* = 0.008) as well as greater QRS-T angle (64° vs. 41° *p* = 0.03) were observed in patients with LVNC in comparison with the healthy control subjects.

Adverse outcomes were observed in 13 patients; among DCM patients, six qualified for a heart transplant, two of them died while awaiting heart transplant, ventricular arrhythmias were observed in four patients, and two of them received an ICD. Among HCM patients, malignant ventricular arrhythmias were observed in four patients, five qualified for an ICD implantation, and one qualified for a heart transplant. In the LVNC group, one patient was qualified for a heart transplant. Atrial flutter and atrial fibrillation were not observed in any of the patients, and one of the patients with HCM had a previous history of supraventricular tachycardia.

Significant differences in terms of the QRS-T angle were observed among cardiomyopathy patients with and without the end point of the study (MACE) (Table 2). QRS-T angle was greater among patients with unfavorable outcomes, independent of whether the outcome was an arrhythmic event or heart failure progression (Table 2). In terms of other ECG parameters, greater PQ, QRS, and QTc intervals as well as two or more negative T waves were observed more frequently among patients with MACE than in those who did not meet the end point of the study.

Serum biomarker concentrations of NT-proBNP were significantly higher among patients with MACE (median 3262 vs. 69 ng/mL, *p* < 0.0001). Similarly, NYHA or Ross scale classes were significantly greater among patients with MACE (*p* < 0.001) than in cardiomyopathy patients without adverse outcomes.

**Figure 1:**
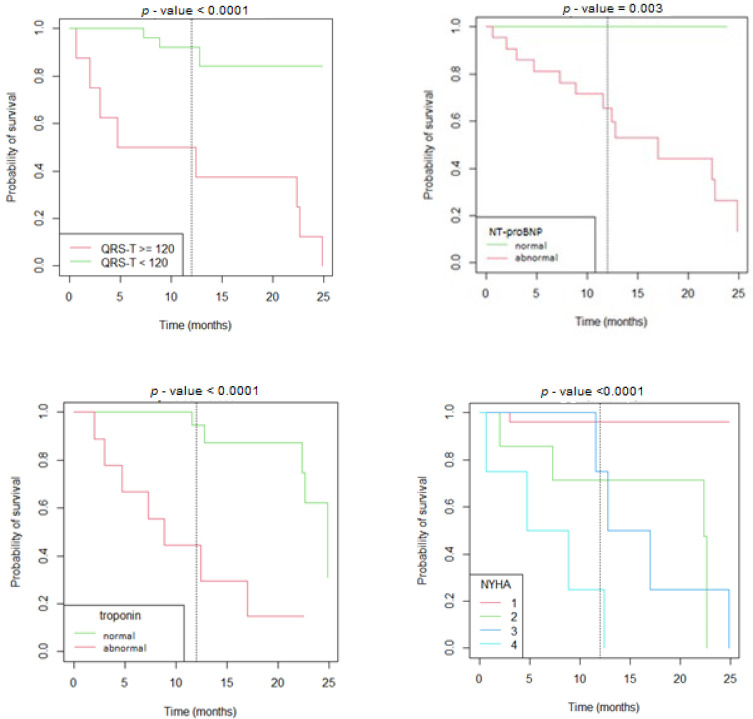
Kaplan–Meier survival curves according to the value of the QRS-T angle, serum concentrations of NT-proBNP and troponin, and NYHA/Ross scale.

In the random forest model, the parameters associated the strongest with adverse outcomes in children with cardiomyopathies were QRS-T angle, NT-proBNP, NYHA or Ross class, and pathological T wave inversions in one or more leads with 92% specificity and 82% sensitivity (Figure S1, Table S2 Supplementary Materials). On Kaplan–Meier curves reduced survival was associated with a QRS-T angle > 120°, greater NYHA class, and abnormal NT-proBNP and troponin I levels (Figure 1).

### 3.1. Dilated Cardiomyopathy

**Figure 2:**
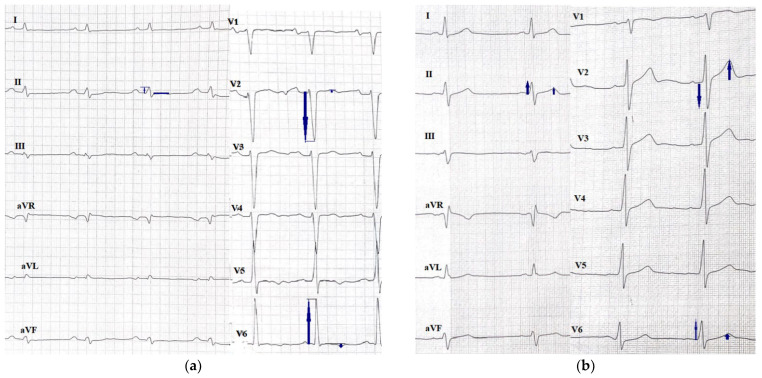
ECGs of patients with DCM with (**a**) an abnormal QRS-T angle of 164° and (**b**) a normal QRS-T angle of 88°.

In the DCM group, the QRS-T angle was significantly greater among patients with unfavorable outcomes (134° vs. 48°, *p* < 0.001) (Table 3); greater QRS-T angle was also observed among DCM patients with malignant arrhythmia (*p* < 0.001) (Table S4 Supplementary Materials) and qualified for a heart transplant (*p* < 0.001) independently. The differences between an ECG with a normal and an abnormal spatial QRS-T angle can be observed in Figure 2. 

Other ECG parameters associated with MACE were greater QTc interval and two or more pathological negative T waves (Table 3). There were no significant differences in terms of PQ interval or QRS duration. There were no significant differences in the ECG parameters (including QRS-T angle) between children with DCM who did not present with MACE and the control group (mean QRS-T 48° vs. 41°, *p* = 0.37). Children with DCM who experienced MACE had significantly greater QRS-T angle, longer QTc interval, and ≥2 pathological negative T waves on their baseline ECG in comparison with the control group (Table S3 Supplementary Materials). 

Concentrations of serum biomarkers (NT-proBNP and troponin) were significantly higher among DCM patients with MACE compared to children with DCM without MACE (NT-proBNP median 3068 vs. 58 pg/mL, troponin median 49 vs. 3 ng/mL) (Table 3). A moderate correlation was observed between QRS-T angle and NT-proBNP (r = 0.55); no significant correlation was found between QRS-T angle and troponin levels. 

In echocardiography, greater LVIDd *z*-scores as well as lower LVPWd *z*-scores and LVEF were observed among patients with unfavorable outcomes (Table 3). A strong positive correlation was observed between LVIDd *z*-score and QRS-T angle (r = 0.72), as well as a moderate correlation between decreased LVEF and greater QRS-T angle (r = −0.69). No significant correlation was found between LVPWd and QRS-T angle. 

In patients with DCM and ventricular tachycardia, a greater QRS duration (85° vs. 70° ms), QRS-T angle (142° vs. 57°), PQ interval (153 vs. 123 ms), and ≥2 pathological negative T waves were observed in comparison with children with DCM without ventricular arrhythmia (Table S4 Supplementary Materials). NT-proBNP was also significantly higher among these patients (2232 vs. 69 pg/mL); however, there were no significant differences in terms of troponin levels (Table S4 Supplementary Materials). Patients with DCM with heart failure end point showed the same risk factors associated with unfavorable outcomes as those with MACE.

The random forest model indicated that the best predictors for MACE among DCM patients were LVEF, NT-proBNP, NYHA/Ross scale, LVIDd z-score, and QRS-T angle.

### 3.2. Hypertrophic Cardiomyopathy

In HCM patients, a greater QRS-T angle was observed in patients with MACE compared to patients without adverse outcomes (133° vs. 85°; *p* = 0.004) (Table 4). HCM patients with MACE had also a greater PQ interval (140 vs. 120 ms, *p* = 0.02) and QRS duration (105 vs. 70 ms, *p* = 0.002), and more frequent presence of two or more pathological negative T waves (*p* = 0.01) (Table 4). 

In comparison with the control group, patients with HCM had a greater QRS-T angle (100° vs. 41°, *p* < 0.001), QTc interval (420 vs. 380 ms, *p* = 0.002), as well as more frequent presence of at least two pathological T-wave inversions (*p* = 0.005). No differences were observed between the HCM group and the control group in terms of heart rate and, durations of PQ or QRS intervals.

Serum concentrations of NT-proBNP were significantly higher among HCM patients with MACE compared to patients without adverse events (2659 vs. 125 pg/mL, *p* = 0.01); the difference in the troponin levels between the two groups was borderline significant (*p* = 0.06) (Table 4). Similarly to DCM patients, higher NYHA or Ross scale was observed among patients with MACE (*p* = 0.038). On correlation analysis, a correlation between NYHA scale and QRS-T angle (r = 0.5) was found, but no significant correlation was found between QRS-T angle and NT-proBNP.

In echocardiography, the IVSd *z*-score was borderline significant in MACE patients (*p* = 0.052). In HCM patients with MACE, there were no significant differences in terms of LVEF or LVIDd in comparison with patients without adverse events.

In HCM patients with ventricular arrhythmia, greater QRS-duration, QRS-T angle and more than two pathological negative T waves were present more frequently in comparison with patients without arrhythmia (Table S5 Supplementary Materials). There were no significant differences in terms of NT-proBNP and troponin levels between HCM patients with and without ventricular tachycardia (Table S5 Supplementary Materials). 

The random forest model showed that QRS, QRS-T angle, LVH and PQ were the best predictors for MACE among HCM patients.

Inter-observer variability according to Lin’s concordance correlation coefficient in terms of QRS-T angle description was 0.99 [CI 0.98; 0.99].

## 4. Discussion

In the present study, we outlined electrocardiographic risk factors associated with unfavorable outcomes among patients with cardiomyopathies. In adults with both dilated and hypertrophic cardiomyopathy, a greater QRS-T angle has been associated with unfavorable outcomes [2,3,4,6]. Similarly, in our study we observed a significantly greater QRS-T angle in patients with MACE, independently of the type of cardiomyopathy. Furthermore, increased QRS-T angle was observed in both heart failure and arrhythmic event groups. The random forest model outlined QRS-T angle as a leading factor in predicting MACE in all cardiomyopathy patients as well as HCM and DCM independently. Kaplan–Meier survival analysis showed that a QRS-T angle >120° was associated with an unfavorable outcome. This suggests that the spatial QRS-T angle could be a helpful parameter in assessing the risk for disease in different types of cardiomyopathies. It appears that the conduction abnormalities on the myocyte level, independently of the cardiomyopathy type, are reflected through the QRS-T angle.

### 4.1. Dilated Cardiomyopathy

In our study, increased QRS-T angle and QTc, as well as the presence of at least two pathologically inverted T waves were associated with MACE in children with DCM. In the scarce studies on children, QTc has been associated with unfavorable outcomes. Chen et al. found a relationship between increased risk of malignant arrhythmia and: longer QRS duration, abnormal T-waves, ST-segment depression, QTc, JTc intervals, and QT and JT dispersion [23]. Similarly, Ture et al. suggested a greater QT interval as well as QT and T-wave peak-end dispersion in children with dilated cardiomyopathy who died [24]. However, none of these studies analyzed the association between the QRS-T angle or ≥2 pathologic negative T-waves and adverse outcomes in children.

In adult patients with idiopathic dilated cardiomyopathy, a relationship between greater QRS-T angle and mortality, risk of an ICD shock or rehospitalization due to heart failure had been observed, with cut-off values ranging between >90° and 152° [2,3,4]. In our cohort, we observed a mean spatial QRS-T angle of 133° among patients with adverse outcomes, which seems to be in agreement with adult reports. The QRS-T angle seems to be a useful parameter in identifying DCM children with the greatest cardiovascular risk, but further studies on a larger number of patients are necessary to confirm our finding. 

In contrast to the study by Chen et al., we found no correlation between pathological T wave inversion in only one ECG lead and adverse outcomes [23]. In our study, at least two negative T waves were associated with MACE. This seems to support the role of the spatial QRS-T angle, because its calculation includes the magnitude of the T-wave in three leads. Thus, pathological inversion of two or more T-waves on a 12-lead ECG could be more specific in identifying patients at greatest risk.

Meulen et al. reported that the serum biomarker NT-proBNP is associated with adverse outcomes in children with dilated cardiomyopathy [25]. A similar observation was made in our study. We found a positive correlation between serum concentrations of NT-proBNP and greater QRS-T angle, which suggests an association between heart failure severity and abnormalities in 12-lead ECG. Troponin I levels were also significantly higher in children with DCM and adverse outcomes, which might indicate another serum biomarker useful in risk stratification. To our knowledge, troponin I levels have not been analyzed previously in the pediatric DCM population. In adults with idiopathic dilated cardiomyopathy, it has been reported that increased NT-proBNP, troponin I, and troponin T levels are associated with increased mortality [26,27,28]. Furthermore, in adults with LMNA mutations, both increased troponin T and NT-proBNP were associated with the presence of malignant arrhythmias [29].

Unsurprisingly, echocardiographic findings such as decreased left ventricular ejection fraction, shortening fraction and increased left ventricular end diastolic dimension have been associated with increased risk of mortality in pediatric dilated cardiomyopathy patients [24,25,30]. In our study, similar observations were made; decreased LVEF and LVPWd, and increased LVIDd *z*-scores were associated with unfavorable outcomes. Correlations between QRS-T angle and echocardiographic parameters such as LVIDd and LVEF were also shown, suggesting that electrocardiographic changes reflect echocardiographic abnormalities and could be helpful in predicting adverse outcomes.

### 4.2. Hypertrophic Cardiomyopathy

Ninety-seven per cent of pediatric HCM patients have abnormalities in their ECG [31]. However, in a multicenter study by Norrish et al. none of the proposed electrocardiographic parameters adequately predicted the risk of malignant arrhythmia in children with HCM [31]. However, in the study by Norrish et al. neither the QRS-T angle nor multiple pathological T waves have been analyzed as parameters associated with unfavorable outcome. In our study, children with ventricular arrhythmias had a significantly greater QRS interval and QRS-T angle (133° vs. 89°), and two or more pathological negative T waves on their baseline ECG, in comparison with children with HCM without ventricular tachycardia. This seems to be in agreement with the study by Cortez et al. on children and adolescents up to 23 years old, in whom spatial QRS-T angle > 124° had a high negative predictive value for excluding risk of ventricular arrhythmias [14]. Thus, both QRS-T angle and multiple negative T wave inversions in ECG might be helpful in identifying patients at greatest risk of adverse outcomes in the pediatric population; these have not been analyzed in larger pediatric cohorts. 

Interestingly, patients with HCM with no MACE also had a significantly greater QRS-T angle than the control group. This is in agreement with a study by Cortez et al. on adults, in which greater QRS-T angle helped to identify patients with HCM [32]. It seems that the QRS-T angle might be helpful in differentiating HCM patients from healthy individuals both in the adult and pediatric populations. Furthermore, because the greatest QRS-T angle was observed among patients with HCM and MACE, regular QRS-T measurements could help to assess the disease’s progression. However, further studies on a larger number of patients are necessary to confirm this hypothesis.

In terms of serum biomarkers, the serum concentration of NT-proBNP was significantly higher among HCM patients with adverse outcomes (2659 vs. 125 pg/mL). This corresponds to the findings by Kaski et al., who showed a relationship between increased NT-proBNP levels and HCM severity among pediatric HCM patients [33]. Thus, we did not find a relationship between malignant arrhythmia occurrence and NT-proBNP in HCM children. This suggests that NT-proBNP may be useful in outlying patients with heart failure progression but not necessarily in those with greatest risk of ventricular arrhythmias. A similar observation was made in an adult study by Coates et al., in which elevated NT-proBNP was a predictor of heart failure death but not of sudden cardiac death [34]. 

CMR plays a significant role in the diagnosis of hypertrophic cardiomyopathy as well as identifying the extent of myocardial fibrosis [35]. Increased troponin I and T levels were associated with adverse cardiovascular events and increased fibrosis on CMR [36,37]. In our study, troponin I levels among HCM patients who had MACE were borderline significant (*p* = 0.06) in comparison with the group without MACE. This may be explained by the fact that the majority of patients with adverse outcomes had arrhythmia and the number of patients who presented with heart failure in this group was low. Increased troponin concentration might be associated more with heart failure progression than with arrhythmia. However, further studies on a larger number of patients are necessary to support this hypothesis.

Correlation analysis in the DCM group showed an association between the QRS-T angle and NT-proBNP as well as QRS-T angle and echocardiographic parameters such as LVEF or LVIDd *z-*score. Thus, in patients with HCM, even though the QRS-T angle was greater among patients with MACE, no correlations between echocardiographic parameters or serum biomarkers were found. Therefore, the QRS-T angle seems to be a valuable parameter in assessing conduction abnormalities, despite their heterogeneous origin. It appears that an increased QRS-T angle may reflect both abnormal stretching of the myocytes and irreversible changes in the myocardium such as fibrosis. On one hand, Li et al. reported in adults a decrease in the QRS-T angle after medical treatment [4], suggesting that some of the myocardial depolarization and repolarization abnormalities may be reversible. On the other hand, Jensen et al. showed a correlation between the QRS-T angle and late gadolinium enhancement on CMR in patients with HCM [38]. This proves that the QRS-T angle is also abnormal in patients with irreversible myocardial changes such as fibrosis. Taking into account the association between QRS-T angle and MACE, it would be worth exploring whether ECG changes precede fibrosis in CMR. Furthermore, in adult patients with HCM it was noted that genotype-positive patients tend to have a greater QRS-T angle than genotype negative patients [32]. Thus, depolarization abnormalities may be associated with the type of genetic mutation. Future studies on a larger population are necessary to approach these questions.

Some limitations of the study can be outlined. Because this was a single-center study and the prevalence of cardiomyopathies in children is low, the included number of patients was limited. Thus, we encourage studies on a larger scale, because the QRS-T angle seems to be an easily obtainable but not well-explored parameter in the pediatric population.

## 5. Conclusions

QRS-T angle appears to be a valuable addition to standard electrocardiogram (ECG) interpretation. It is associated with adverse outcomes in children with both dilated and hypertrophic cardiomyopathies. Furthermore, serum biomarkers such as troponin and NT-proBNP might also be useful in outlining patients at the highest risk of heart failure progression and death.

## Figures and Tables

**Table 1:** Patients’ characteristics.

	DCM *n* = 19	HCM *n* = 17	LVNC *n* = 6	Control *n* = 19	*p*-Value
gender (*n* female/male)	10/9	8/9	5/1	10/9	0.49
Age (years)	8 (3–14)	10 (3–16.5)	10.5 (8–15.5)	8 (4–15)	0.73
BSA (m^2^)	1.1 (0.6–1.8)	1.5(0.6–1.9)	1.1(0.9–1.3)	1.2(0.7–1.7)	0.85
observation time (months)	14 ± 8	11 ± 7	12 ± 8	n/a	0.42

Data are presented as median and IQR in parenthesis or mean and standard deviation (±), *n*—number of patients; DCM—dilated cardiomyopathy, HCM—hypertrophic cardiomyopathy, LVNC—left ventricular non-compaction, BSA—body surface area, n/a—not assigned.

**Table 2:** QRS-T angle in cardiomyopathy patients according to the adverse outcome.

	QRS-T Angle		
	No End Point	Met End Point	*p*-Value
VT	70.1° ± 31.8°	137.2° ± 25.3°	<0.001
ICD	72.5° ± 34.5°	135.6° ± 24.2°	<0.001
HTx	74.7° ± 36.6°	133.7° ± 22.8°	<0.001
All end points	64.7° ± 28°	133.2° ± 23°	<0.001

Data are given as mean and standard deviation, VT—ventricular tachycardia, ICD—implantable cardioverter defibrillator inserted/qualified for insertion, HTx—qualification for heart transplant.

**Table 3:** Parameters associated with adverse outcomes in patients with DCM.

	DCM no MACE *n* = 13	DCM MACE *n* = 6	*p*-Value
HR (bpm)	88 (82–100)	100 (74–137)	0.759
PQ (ms)	123.8 ± 18.5	140.0 ± 28.9	0.157
QRS (ms)	76.2 ± 10.4	85.0 ± 18.7	0.317
QTc (ms)	392.9 ± 31.4	431.1 ± 38.9	**0.035**
QRS-T (degrees)	48.2 ± 22.9	133.7 ± 22.8	**<0.001**
Negative T-waves ≥ 2 leads	0	4 (66%)	**0.007**
NT-proBNP (pg/mL)	58 (37–146)	3068 (2123–3810)	**<0.001**
Troponin (ng/mL)	2.7 (1.5–9.5)	48.9 (25.9–95.4)	**0.013**
NYHA/Ross (I/II/III/IV)	13 (100%)/0/0/0	0/1 (17%)/1 (17%)/4 (66%)	**<0.001**
LVEF %	44.4 ± 6.78	21.9 ± 5.78	**<0.001**
LVIDd *z-*score	2.86 ± 0.87	5.50 ± 0.77	**<0.001**
LVPWD *z*-score	0.33 ± 0.493	1.07 ± 0.66	**0.042**

*n* number of patients, DCM—dilated cardiomyopathy, MACE—major adverse cardiac event, HR heart rate (in beats per minute), ms—milliseconds, LVEF—left ventricular ejection fraction, LVIDd—left ventricular internal diastolic diameter, LVPWd left ventricular posterior wall thickness in diastole.

**Table 4:** Parameters associated with adverse outcomes in children with HCM.

	HCM no MACE *n* = 11	HCM MACE *n* = 6	*p*-Value
HR (bpm)	97.4 ± 26.98	73.8 ± 17.72	**0.048**
PQ (ms)	120 (120–135)	140 (140–163)	**0.021**
QRS (ms)	70 (70–70)	105 (85–118)	**0.002**
QTc (ms)	406.9 ± 27.46	433.6 ± 34.32	0.1
QRS-T (degrees)	84.6 ± 25.69	132.5 ± 26	**0.004**
Negative T-waves ≥ 2 leads	2 (18%)	5 (100%)	**0.012**
ST elevation	0	3 (50%)	0.055
ST depression	1 (9%)	3 (50%)	0.193
LVH	0 (0%)	5 (83%)	0.416
NT-proBNP (pg/mL)	124.5 (45–908)	2659 (1611–4894)	**0.011**
Troponin (ng/mL)	8.7 (1.5–26.4)	50.1 (24.4–131.1)	0.06
NYHA/Ross (I/II/III/IV)	8 (73%)/3 (27%)/0/0	1 (17%)/3 (50%)/2 (33%)/0	**0.038**
LVEF %	61.9 ± 6.73	51.7 ± 19.01	0.253
LVIDD *z-*score	−1.87 ± 1.576	−1.74 ± 2.471	0.909
LVPWD *z*-score	2.90 (2.15–4.1)	6.40 (2.85–10.25)	0.191
IVSd *z-*score	7.4 ± 4.4	12.6 ± 4.7	0.052

*n* number of patients, HCM hypertrophic cardiomyopathy, MACE—major adverse cardiac event, HR heart rate (in beats per minute), LVEF- left ventricular ejection fraction, LVIDd—left ventricular internal diastolic diameter, LVPWd—left ventricular posterior wall thickness in diastole, IVSd—interventricular septum in diastole, LVH—left ventricular hypertrophy.

## Supplementary Materials

The following supporting information can be downloaded at: https://www.mdpi.com/article/10.3390/jcm11236930/s1, Table S1: Differences in the baseline ECG, echocardiographic parameters, adverse events occurrence among HCM, DCM and LVNC patients; Table S2: Random forest regression model in children with cardiomyopathies—specificity and sensitivity; Figure S1. Random forest model presenting variables associated with unfavorable outcomes in children with cardiomyopathies; Table S3: Comparison between DCM patients with MACE and control group; Table S4: Comparison between DCM patients with and without ventricular tachycardia; Table S5: Comparison between HCM patients with and without ventricular tachycardia.
