# Supplementary material for: Electrocardiographic Parameters Associated with Adverse Outcomes in Children with Cardiomyopathies
Source: J Clin Med. 2022 Nov 24;11(23):6930. doi: 10.3390/jcm11236930 (PMC9738383; doi:10.3390/jcm11236930)
Supplement: Supplementary file 1 [file jcm-11-06930-s001.zip › jcm-1998152-supplementary.pdf]

Supplementary materials

Table S1. Differences in the baseline ECG, echocardiographic parameters, adverse events occurrence among HCM, DCM and LVNC patients.

	DCM	HCM	LVNC	p-value
HR [bpm]	97.2 ± 28.89	89.1 ± 26.22	79.0 ± 23.07	0.337
PQ [ms]	128.9 ± 22.83	135.3 ± 23.48	133.3 ± 16.33	0.693
QRS [ms]	80 (70-80)	70 (70-100)	85 (80-98)	0.241
QRS-T [º]	75.2 ± 46.50	99.6 ± 33.86	64.1 ± 16.99	0.107
QTc_mean [ms]	404.9 ± 37.56	416.3 ± 31.80	420.9 ± 22.43	0.471
LA enlargement (*n)*	2 (12%)	3 (16%)	1 (17%)	0.817
RA enlargement (*n)*	0	3 (18%)	2 (33%)	0.057
RVH (*n*)	0	0	0	X
LVH (*n*)	2 (11%)	5 (29%)	0	0.157
Fragmented QRS (*n*)	8 (42%)	5 (29%)	1 (20%)	0.563
Negative T-wave ≥2 leads (*n*)	4 (21%)	7 (44%)	0 (0%)	0.113
RBBB (*n*)	0	0	1 (16.7%)	**0.046**
LBBB (*n*)	1 (5%)	1 (6%)	0	0.836
LVEF (%)	40 (12 – 47)	61 (21 – 65)	55.5 (51 – 56)	**< 0.001**
LVIDd *z*-score	3.70 ± 1.50	-1.82 ± 1.86	0.75 ± 0.73	**< 0.001**
LVWPD *z*-score	0.57 (0.14 – 0.92)	3.40 (2.00 – 5.40)	1.05 (0.03 – 1.55)	**< 0.001**
IVSd *z*-score	0.57 ± 0.69	9.20 ± 5.06	0.50 ± 1.01	**< 0.001**
VT (*n*)	4 (21%)	4 (24%)	0 (0%)	0.431
ICD (*n*)	2 (11%)	5 (29%)	0 (0%)	0.157
HTx (*n*)	6 (32%)	1 (6%)	1 (17%)	0.145
LVAD (*n*)	3 (16%)	0 (0%)	0 (0%)	0.142
Death (*n*)	2 (11%)	0 (0%)	0 (0%)	0.281
All end points (*n*)	6 (32%)	6 (35%)	1 (17%)	0.696

Data are presented as median and IQR in parenthesis or mean and standard deviation (±) or percentages, *n* – number of patients; DCM- dilated cardiomyopathy, HCM – hypertrophic cardiomyopathy, LVNC – left ventricular non-compaction, HR – heart rate (in beats per minute), ms – milliseconds, LA – left atrium, RA – right atrium, RVH – right ventricular hypertrophy, LVH left ventricular hypertrophy, RBBB -right bundle branch block, LBBB- left bundle branch block, LVEF- left ventricular ejection fraction, LVIDd - left ventricular internal diastolic diameter, LVPWd left ventricular posterior wall thickness in diastole, VT – ventricular tachycardia, ICD - implantable cardioverter defibrillator qualification/implantation, HTx- heart transplant qualification, LVAD – left ventricular assist device implantation

Table S2. Random Forest regression model in children with cardiomyopathies – specificity and sensitivity

	ACC	sensitivity	specificity	NPV	PPV	AUC (95%CI)
Random forest	89.5%	81.8%	92.59%	81.81%	92.59%	0.87 (0.74; 1.00)

NPV – negative predictive value, PPV positive predictive value, AUC – area under the curve

Figure S1. Random Forest model presenting variables associated with unfavorable outcomes in children with cardiomyopathies

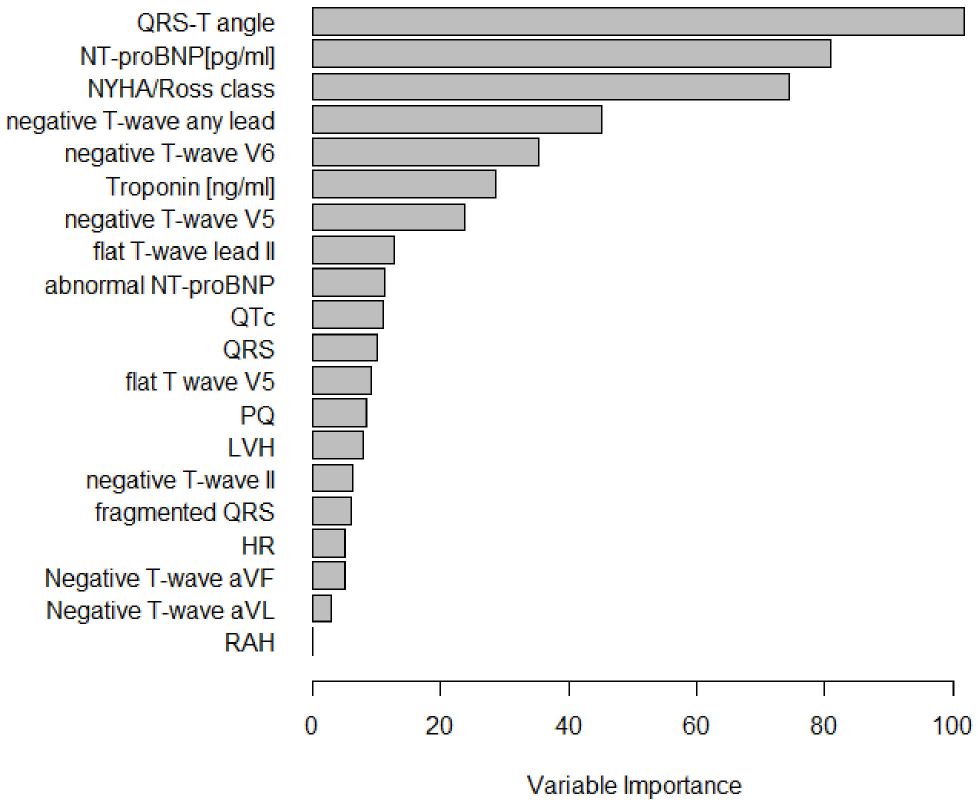

Table S3. Comparison between DCM patients with MACE and control group

	DCM with MACE *n=6*	Control group *n*=19	p-value
HR [bpm]	100 (74 – 137)	77 (68 – 99)	0.373
PQ [ms]	0.14 ± 0.03	0.13 ± 0.02	0.323
QRS [ms]	0.09 ± 0.02	0.08 ± 0.01	0.296
QTc_mean [ms]	0.43 ± 0.04	0.38 ± 0.02	**0.031**
QRS-T [º]	133.7 ± 22.82	40.6 ± 23.22	**<0.001**
Negative T-wave 1 lead<br>0<br>1	6 (100%)<br>0	17 (90%)<br>2 (10%)	1
Negative T-wave ≥2 leads<br>0<br>1	2 (33%)<br>4 (67%)	19 (100%)<br>0	**0.001**
ST depression<br>0<br>1	6<br>0	19 (100%)<br>0	X

ST elevation<br>0<br>1	5 (83%)<br>1 (17%)	19 (100%)<br>0	0.534
LVEF (%)	27.9 ± 5.78	62.2 ± 6.17	**< 0.001**
LVEDD *z*-score	5.50 ± 0.77	0.17 ± 0.68	**< 0.001**
LVPWd *z*-score	1.07 ± 0.66	-0.32 ± 0.93	**0.002**
IVSd z-score	1.3 (0.9 – 1.4)	0.3 (-0.7 – 0.9)	0.06

Data are presented as median and IQR in parenthesis or mean and standard deviation (±) or percentages 0/1 – absent/present, *n* – number of patients; DCM- dilated cardiomyopathy, MACE – major adverse cardiac event, HR – heart rate (in beats per minute), ms – milliseconds, LVEF- left ventricular ejection fraction, LVIDd - left ventricular internal diastolic diameter, LVPWd left ventricular posterior wall thickness in diastole, IVSd - interventricular septum in diastole

Table S4. Comparison between DCM patients with and without ventricular tachycardia

	no VT<br>*n*=15	VT<br>*n*=4	p-value
HR [bpm]	97 (83.5 – 110.5)	79.5 (64.2 – 95)	0.294
QRS [ms]	70 (70 – 80)	85 (80 – 97.5)	**0.035**
QTc [ms]	401.6 ± 39.02	417.4 ± 33.15	0.471
PQ [ms]	122.7 ± 18.31	152.5 ± 25.00	**0.015**
QRS-T [º]	57.42 ± 32.58	141.73 ± 23.18	**<0.001**
Negative T-waves 1 lead<br>0<br>1	14 (93%)<br>1 (7%)	4 (100%)<br>0	1
Negative T-waves ≥2 leads<br>0<br>1	14 (93%)<br>1 (7%)	1 (25%)<br>3 (75%)	**0.022**
ST depression<br>0<br>1	15 (100%)<br>0	4 (100%)<br>0	-
ST elevation<br>0<br>1	15 (100%)<br>0	3 (75%)<br>1 (25%)	0.466
NT-proBNP [pg/ml]	69 (39 – 286)	2232 (1958 – 2799)	**0.02**
Troponin [ng/ml]	5.1 (1.5 –18.1)	25.9 (13.9 – 60.7)	0.234
Ross/NYHA class<br>1<br>2<br>3<br>4	13 (87%)<br>0<br>0<br>2 (13%)	0<br>1 (25%)<br>1 (25%)<br>2 (50%)	**0.005**
LVEF (%)	41.4 ± 10.1	21.9 ± 7.28	**0.004**
LVIDd *z*-score	3.11 ± 1.04	5.90 ± 0.54	**<0.001**

LVPWd *z*-score	0.47 ± 0.595	0.93 ± 0.749	0.32
IVSd *z*-score	0.30 (-0.05 – 1)	1.30 (0.94 – 1.32)	0.292

Data are presented as median and IQR in parenthesis or mean and standard deviation (±), *n* – number of patients; 0/1 – absent/present, DCM- dilated cardiomyopathy, VT-ventricular tachycardia, HR – heart rate (in beats per minute), ms – milliseconds, LVEF- left ventricular ejection fraction, LVIDd - left ventricular internal diastolic diameter, LVPWd left ventricular posterior wall thickness in diastole, IVSd - interventricular septum in diastole

Table S5. Comparison between HCM patients with and without ventricular tachycardia

	no VT<br>*n*=13	VT<br>*n*=4	p-value
HR/min	85 (75 – 115)	66.5 (57.5 – 79.8)	0.1
QRS [ms]	70 (70 – 80)	105 (95 – 113)	**0.019**
QTc [ms]	413.7 ± 32.78	425.0 ± 31.08	0.552
PQ [ms]	130 (120 – 140)	140 (140 – 148)	0.149
QRS-T [º]	88.6 ± 28.07	132.6 ± 30.02	**0.018**
Negative T wave in 1 lead<br>0<br>1	 10 (83%)<br>2 (17%)	 4 (100%)<br>0	1
Negative T-wave ≥2 leads<br>0<br>1	 9<br>3	 0<br>4	**0.042**
ST depression<br>0<br>1	 11<br>2	 2<br>2	0.451
ST elevation<br>0<br>1	 12<br>1	 2<br>2	0.234
NT-proBNP [pg/ml]	134 (63 – 2588)	1760 (1317 – 2358)	0.317
Troponin I [ng/ml]	14.4 (2.9 – 48.2)	86.9 (15.4 – 338.1)	0.227
Ross/NYHA class<br>1<br>2<br>3<br>4	 8<br>4<br>1<br>0	 1<br>2<br>1<br>0	0.392
LVEF %	61 (54 – 65)	61 (50 – 63)	0.733
LVIDd z-score	-1.45 ± 1.76	-3.03 ± 1.88	0.202
LVPWd z-score	2.9 (2.0 – 4.5)	9.3 (5.9 – 11.6)	0.141
IVSd z-score	8.7 ± 5.56	10.8 ± 3	0.369

Data are presented as median and IQR in parenthesis or mean and standard deviation (±), *n* – number of patients; 0/1 – absent/present, HCM- hypertrophic cardiomyopathy, VT-ventricular tachycardia, HR – heart rate (in beats per minute), ms – milliseconds, LVEF- left ventricular ejection fraction, LVIDd - left ventricular internal diastolic diameter, LVPWd left ventricular posterior wall thickness in diastole, IVSd - interventricular septum in diastole
